# Cultural competence in forensic psychiatry

**DOI:** 10.1192/j.eurpsy.2025.242

**Published:** 2025-08-26

**Authors:** K. Goethals

**Affiliations:** Antwerp University Hospital, Antwerp, Belgium

## Abstract

**Abstract:**

In this paper several issues about cultural competence will be addressed, such as assumptions, Mason’s five progressive steps and learning needs of professionals. The notion of cultural competence combines an understanding of different belief systems, good communication skills (including highly specialist skills such as the communication of internal emotional states, and the cultural adaptation of treatment models and therapies. Professionals can measure theircompetence on a continuum developed by James Mason. His five progressive steps are: cultural destructiveness, incapacity, blindness, pre-competence, and competence. Next, the mental health needs of refugees will be discussed, especially for those who are at risk to become violent offenders. For example, some types of environmental and psychosocial stressors that refugees may experience day-to-day. Some of the cultural and attitudinal factors should be taken into account when working with refugees and wider communities. Finally, educational needs for trainees in (forensic) psychiatry and (forensic) psychiatrists will be highlighted. Knowledge about culture, ethnicity, race, religion, and identity is hereby crucial. Reflections will be made on the presented case.

**Disclosure of Interest:**

None Declared

